# Biomarker‐Based Diagnosis and Care Pathways for Alzheimer's Disease in the Era of Disease‐Modifying Treatments: A Consensus Statement by Belgian Experts

**DOI:** 10.1111/ene.70668

**Published:** 2026-07-31

**Authors:** Tim Van Langenhove, Sara Van Mossevelde, Marijke Miatton, Jan De Lepeleire, Rose Bruffaerts, Jean Christophe Bier, Kurt Segers, Jurn Verschraegen, Mirko Petrovic, Manfredi Ventura, Haroun Jedidi, Ingo Beyer, Eric Mormont, Rik Vandenberghe, Aline Delva, Christian Gilles, Donatienne Van Weehaeghe, Gaëtane Picard, Peter De Deyn, Mélanie Strauss, Evert Thiery, Eric Salmon, Gert Cypers, Joris Vlaemynck, Katrin Gillis, Jan Versijpt, Maria Bjerke, Anne Sieben, Bernard Hanseeuw, Sebastiaan Engelborghs, Olivier Deryck, the BeDeCo members

**Affiliations:** ^1^ Cognitive Center, Department of Neurology Ghent University Hospital Ghent Belgium; ^2^ Department of Neurology University Hospital of Antwerp Antwerp Belgium; ^3^ Translational Neurosciences, Faculty of Medicine and Health Sciences University of Antwerp Antwerp Belgium; ^4^ Department of Public Health and Primary Care, Academic Center for General Practice KU Leuven Leuven Belgium; ^5^ Computational Neurology, Experimental Neurobiology Unit (ENU), Department of Biomedical Sciences University of Antwerp Antwerp Belgium; ^6^ Biomedical Research Institute Hasselt University Hasselt Belgium; ^7^ Department of Neurology, Hôpital Universitaire de Bruxelles (H.U.B), CUB Hôpital Érasme Université Libre de Bruxelles Brussels Belgium; ^8^ Department of Neurology and Geriatrics Brugmann University Hospital Brussels Belgium; ^9^ Expertisecentrum Dementie Vlaanderen vzw Antwerp Belgium; ^10^ Department of Internal Medicine and Paediatrics Ghent University Ghent Belgium; ^11^ Department of Neurosciences Grand Hôpital de Charleroi Charleroi Belgium; ^12^ Department of Neurology Valdor Hospital Liège Belgium; ^13^ Geriatric Hospital Scheutbos (Silva Medical) Brussels Belgium; ^14^ Frailty in Ageing Research Group (FRIA) Vrije Universiteit Brussel (VUB) Brussels Belgium; ^15^ Department of Geriatrics Universitair Ziekenhuis Brussel Brussels Belgium; ^16^ Department of Neurology CHU UCL Namur Yvoir Belgium; ^17^ Institute of Neuroscience (IoNS) Université Catholique de Louvain Brussels Belgium; ^18^ Department of Neurology University Hospitals Leuven Leuven Belgium; ^19^ Cognitive and Behavioral Geriatrics Vivalia Libramont‐Chevigny Belgium; ^20^ Department of Radiology and Nuclear Medicine Ghent University Hospital Ghent Belgium; ^21^ Department of Neurology Clinique Saint‐Pierre Ottignies Belgium; ^22^ Department of Neurology, Memory Clinic Antwerp Ziekenhuis aan de Stroom Antwerp Belgium; ^23^ Neurochemistry and Behaviour Group, Experimental Neurobiology Unit, Department of Biomedical Sciences University of Antwerp Antwerp Belgium; ^24^ Laboratory of Experimental Neurology Université libre de Bruxelles (ULB) Brussels Belgium; ^25^ Department of Neurology Ghent University Hospital Ghent Belgium; ^26^ Memory Clinic, Department of Neurology Centre Hospitalier Universitaire (CHU) Liège Liège Belgium; ^27^ Giga‐CRC‐IVI Liège University Liège Belgium; ^28^ Center for Neurology and Cognitive Disorders Aalst Belgium; ^29^ Department of Geriatrics, Centre for Cognitive Disorders AZ Sint‐Jan Brugge AV Brugge Belgium; ^30^ Centre for Research and Innovation in Care University of Antwerp Wilrijk Belgium; ^31^ Department of Neurology and Bru‐BRAIN Universitair Ziekenhuis Brussel Brussels Belgium; ^32^ Neuroprotection and Neuromodulation (NEUR) Research Group, Center for Neurosciences (C4N) Vrije Universiteit Brussel Brussels Belgium; ^33^ Department of Clinical Biology Universitair Ziekenhuis Brussel Brussels Belgium; ^34^ Department of Pathology University Hospital of Antwerp Antwerp Belgium; ^35^ IBB‐NeuroBiobank Born Bunge Institute Antwerp Belgium; ^36^ Department of Neurology Cliniques Universitaires St Luc Brussels Belgium; ^37^ Department of Neurology AZ St‐Lucas Brugge Belgium

**Keywords:** Alzheimer's disease, biomarkers, care pathways, diagnosis, disease‐modifying therapy

## Abstract

**Background:**

The recent approval of disease‐modifying therapies (DMTs) for early Alzheimer's disease (AD) marks a major shift in clinical practice. Biomarker confirmation of amyloid pathology is now required alongside clinical assessment, and blood‐based tests are improving accessibility. This creates increased demand for timely and accurate diagnosis while avoiding overdiagnosis in low‐probability cases. This Belgian consensus aims to guide biomarker‐based diagnosis of AD in the era of DMTs and to highlight the system adaptations required for safe and equitable implementation. Belgium, with universal healthcare but regionally organised dementia care, provides a relevant case to illustrate both opportunities and challenges.

**Methods:**

This consensus was developed by 31 experts in cognitive neurology, geriatrics, neuropsychology, neuroimaging, neurochemistry, and primary care, coordinated by the Belgian Dementia Council (BeDeCo). Recommendations were based on multidisciplinary discussion, current evidence, and the organisation of dementia care in Belgium.

**Results:**

The consensus outlines a stepwise diagnostic approach that integrates clinical assessment with biomarker confirmation using cerebrospinal fluid, amyloid‐PET, and emerging blood‐based tests. We review the strengths and limitations of each modality and provide guidance for use across clinical scenarios. Using Belgium as a case example, we illustrate challenges that are shared across European healthcare systems, such as limited reimbursement, unequal access to expertise, and insufficient diagnostic capacity, and formulate pragmatic recommendations to address these issues.

**Conclusions:**

This consensus offers practical guidance for embedding biomarker‐based diagnostic strategies into clinical care. By outlining structured pathways and system‐level priorities, it facilitates safe, feasible, and equitable implementation of DMTs for AD.

## 1 Introduction

Alzheimer's disease (AD) is the leading cause of dementia and represents an urgent and growing public health challenge in ageing societies [1]. In Belgium, over 200,000 people are estimated to be living with dementia, most of them due to AD [2]. Across the European Union, around 7 million people are currently affected, a number projected to more than double by 2050 [3]. Dementia is a major contributor to mortality in older adults, with rising impact in recent decades. The economic burden is substantial, driven by healthcare use, informal care, institutional care, and productivity losses, with annual societal costs in Western Europe estimated at nearly €40,000 per person [4]. To support clarity, key terms such as the AD continuum, biomarker categories, and disease‐modifying treatments (DMTs) are defined in Table 1 [5, 6].

**TABLE 1:** Important terms and definitions for Alzheimer's disease and disease‐modifying therapies.

Dementia	A syndrome defined by acquired and measurable decline in one or more areas of thinking ability (such as memory, language, attention, or problem solving) that interferes with independence in everyday life.
Mild cognitive impairment (MCI)	A syndrome defined by acquired and measurable decline in one or more areas of thinking ability, greater than expected for age, but without loss of independence in daily activities.
Alzheimer's disease (AD)	The leading cause of dementia, defined by amyloid‐β plaques and hyperphosphorylated tau tangles in the brain.
Early‐stage Alzheimer's disease	Clinical stage including MCI due to AD and mild AD dementia (cognitive impairment affecting complex but not basic activities of daily living, for example, managing finances while self‐care is preserved).
Amyloid beta (Aβ)	Peptide derived from amyloid precursor protein (APP), which can aggregate into extracellular plaques in the brain.
Tau	Protein that stabilises nerve cells and that, when abnormally hyperphosphorylated, forms tangles inside neurons.
Disease‐modifying therapy (DMT)	A treatment aimed at slowing or stopping the underlying disease process, rather than only alleviating symptoms.
Cerebrospinal fluid (CSF)	Clear fluid surrounding the brain and spinal cord, in which certain proteins can be measured to indicate Alzheimer's pathology.
Positron emission tomography (PET)	An imaging method using radioactive tracers to show brain processes. FDG‐PET measures glucose metabolism to detect reduced brain activity, while amyloid and tau‐PET detect abnormal protein deposits.
Amyloid‐related imaging abnormalities (ARIA)	MRI findings such as fluid accumulation or microbleeds that can occur during treatment with anti‐amyloid drugs.
Apolipoprotein E gene (*APOE*)	A gene involved in fat transport. Three common variants exist: ε2, ε3, and ε4. The ε4 variant increases the risk of AD and ARIA with anti‐amyloid treatments.
Biomarker	A measurable biological characteristic (e.g., in blood, cerebrospinal fluid, or imaging) that reflects normal physiology, disease processes, or responses to treatment.

The recent approval of DMTs targeting amyloid pathology marks an important milestone for patients and families [7, 8]. On April 15, 2025, the European Commission approved lecanemab (Leqembi) for the treatment of early symptomatic AD, defined as mild cognitive impairment (MCI) due to AD or mild AD dementia. On July 25, 2025, donanemab (Kisunla) received a positive opinion from the Committee for Medicinal Products for Human Use (CHMP) for the same indication. In Europe, treatment is restricted to individuals who are noncarriers or heterozygotes for the apolipoprotein E (*APOE*) ε4 gene variant, as ε4 homozygotes have a markedly higher risk of amyloid‐related imaging abnormalities (ARIA) observed in clinical trials [7, 8]. Additional exclusion criteria include prior intracerebral haemorrhage, more than four cerebral microbleeds, superficial siderosis, or ongoing anticoagulant therapy. Baseline and regular MRI monitoring are mandatory, and treatment must be initiated and supervised by physicians experienced in AD. An overview of approved and emerging treatments for AD in Europe is provided in Table 2.

**TABLE 2:** Overview of current treatment strategies for Alzheimer's disease in Europe.

	Cholinesterase inhibitors	Memantine	Lecanemab (Leqembi)	Donanemab (Kisunla)	Cognitive rehabilitation
Type	Symptomatic pharmacological	Symptomatic pharmacological	Disease modifying therapy	Disease modifying therapy	Non‐pharmacological intervention
Mechanism	Cholinesterase inhibition	NMDA receptor antagonist	Anti‐amyloid monoclonal antibody	Anti‐amyloid monoclonal antibody	Cognitive function training
Indication	Mild to moderate AD	Moderate to severe AD	MCI due to AD, and mild AD	MCI due to AD, and mild AD	MCI due to AD, and mild to moderate AD
Biomarker requirements	None	None	Amyloid positivity + APOE genotyping	Amyloid positivity + APOE genotyping	None
Monitoring requirements	Clinical follow‐up	Clinical follow‐up	MRI monitoring for ARIA and clinical follow‐up	MRI monitoring for ARIA and clinical follow‐up	Clinical follow‐up
Effectiveness	Average improvement of 2–3 points on ADAS‐Cog scale over 6–12 months; small additional benefits on behaviour and daily functioning.	Average improvement of 1–2 points on ADAS‐Cog scale; small additional benefits on behaviour and daily functioning.	Primary outcome: difference in CDR‐SB of 0.45 points at 18 months.	Key secondary outcome: difference in CDR‐SB of 0.67 points at 18 months.	Small to moderate effect sizes on cognitive scores and daily activities, depending on intensity and duration.
Side effects	Gastrointestinal symptoms (nausea, vomiting, diarrhoea) in 10%–30% of patients; headache, muscle cramps, nightmares, and bradycardia less common.	Generally well tolerated; dizziness, headache, constipation, and confusion uncommon.	Any ARIA reported in 21.5% of patients, generally asymptomatic; infusion reactions in 26.4%.	Any ARIA reported in 36.8% of patients with the original dosing scheme, generally asymptomatic; lowered to 23.6% with the modified titration scheme. Infusion reactions in 8.7%.	Generally well tolerated; no reported adverse effects.
EMA approval	EMA approved 1997–2000	EMA approved 2002	EMA approved April 2025	EMA approved September 2025	Guideline‐endorsed
Reimbursement status (Belgium, as of early 2026)	Reimbursed	Reimbursed	Not reimbursed	Not reimbursed	Partially reimbursed

*Note:* *Ginkgo biloba* is licenced as a symptomatic treatment for mild to moderate dementia in Germany and some other European countries, but evidence remains inconsistent, and it is not regarded as an evidence‐based therapy for AD in most international guidelines.

The AD treatment landscape continues to evolve rapidly, with more than 140 agents currently in clinical trials, targeting multiple pathological mechanisms including amyloid, tau, inflammation, and synaptic dysfunction [9].

The arrival of DMTs brings not only new opportunities but also major challenges for clinical practice and healthcare organisation. Accurate and timely diagnosis is more important than ever [10]. Appropriate use in turn requires not only careful clinical evaluation and biomarker confirmation of amyloid pathology, but also a healthcare system prepared to deliver early, safe, and equitable access to treatment.

This statement was developed by 31 experts in cognitive neurology, geriatrics, neuropsychology, neuroimaging, neurochemistry, and primary care, coordinated by the Belgian Dementia Council (BeDeCo). The coordinating authors drafted the framework and identified key topics from a review of current evidence. Topic‐specific sections were assigned to domain experts, and drafts were refined through iterative review rounds with written input from all members until consensus was reached. No recommendation was excluded from the final statement because of unresolved disagreement. All authors approved the final version and recommendations as formulated in Box 1. The aim of this consensus is to provide practical guidance on biomarker‐based diagnosis of AD in the era of emerging DMTs and to support clinicians, policymakers, researchers, and administrators in preparing healthcare systems for their safe and equitable use. Recent international frameworks have addressed the diagnostic evaluation process in primary and specialist care [11], recommended biomarker strategies for the aetiological diagnosis of neurocognitive disorders [12] and reviewed the evolving diagnostic landscape [13]. This consensus integrates these into a single stepwise pathway from clinical assessment through biomarker‐based diagnosis to DMT eligibility, incorporates plasma pTau217 as a triage tool within specialist memory clinics, and addresses healthcare system adaptations required for implementation. While Belgium serves as the primary case example, the principles and pathway are designed to be applicable across European and other healthcare systems.

BOX 1 **Key recommendations for biomarker‐based diagnosis and care pathways in Alzheimer's disease (Belgian consensus).**

- Clinical context first: Perform biomarker testing only in individuals with objective cognitive decline and a reasonable suspicion of AD. Amyloid positivity alone does not establish a clinical diagnosis of AD.
- Stepwise approach: Begin in primary care with clinical evaluation, followed by stepwise specialist assessment and biomarker confirmation.
- MRI is essential: Required for differential diagnosis and ARIA safety screening prior to initiation of anti‐amyloid treatment.
- Amyloid confirmation: CSF Aβ42/Aβ40 + pTau (181/217) + tTau; use amyloid‐PET to define amyloid status if lumbar puncture is not feasible or results are inconclusive.
- Blood‐based biomarkers: Use plasma pTau217 (or pTau217/Aβ42) as a triage tool in memory clinics with access to all other required diagnostic tests, applying validated assays with uniformly defined cut‐offs; confirm indeterminate results with CSF or amyloid‐PET.
- *APOE* genotyping and counselling: Mandatory before anti‐amyloid therapy but not otherwise indicated, with pre‐ and post‐test counselling provided by a genetics professional or relevant specialist (neurologist, psychiatrist, or geriatrician).
- Ethical principles: Ensure appropriate counselling, informed consent, and sensitive disclosure of biomarker and genetic results at each stage of the diagnostic pathway.
- System readiness: Expand and formally recognise memory clinics, ensure regional distribution, and provide structural reimbursement for neuropsychological assessment, blood‐based biomarkers, CSF analysis, FDG‐ and amyloid‐PET imaging.
- Future integration: Incorporate clinically validated biomarkers into care pathways, prioritising blood‐based tests while updating PET indications as evidence evolves.
- Equitable access: Apply uniform criteria and reimbursement policies to avoid inequalities in diagnosis and treatment, including across diverse populations.

## 2 The Clinical and Biological Spectrum of Alzheimer's Disease

**FIGURE 1:**
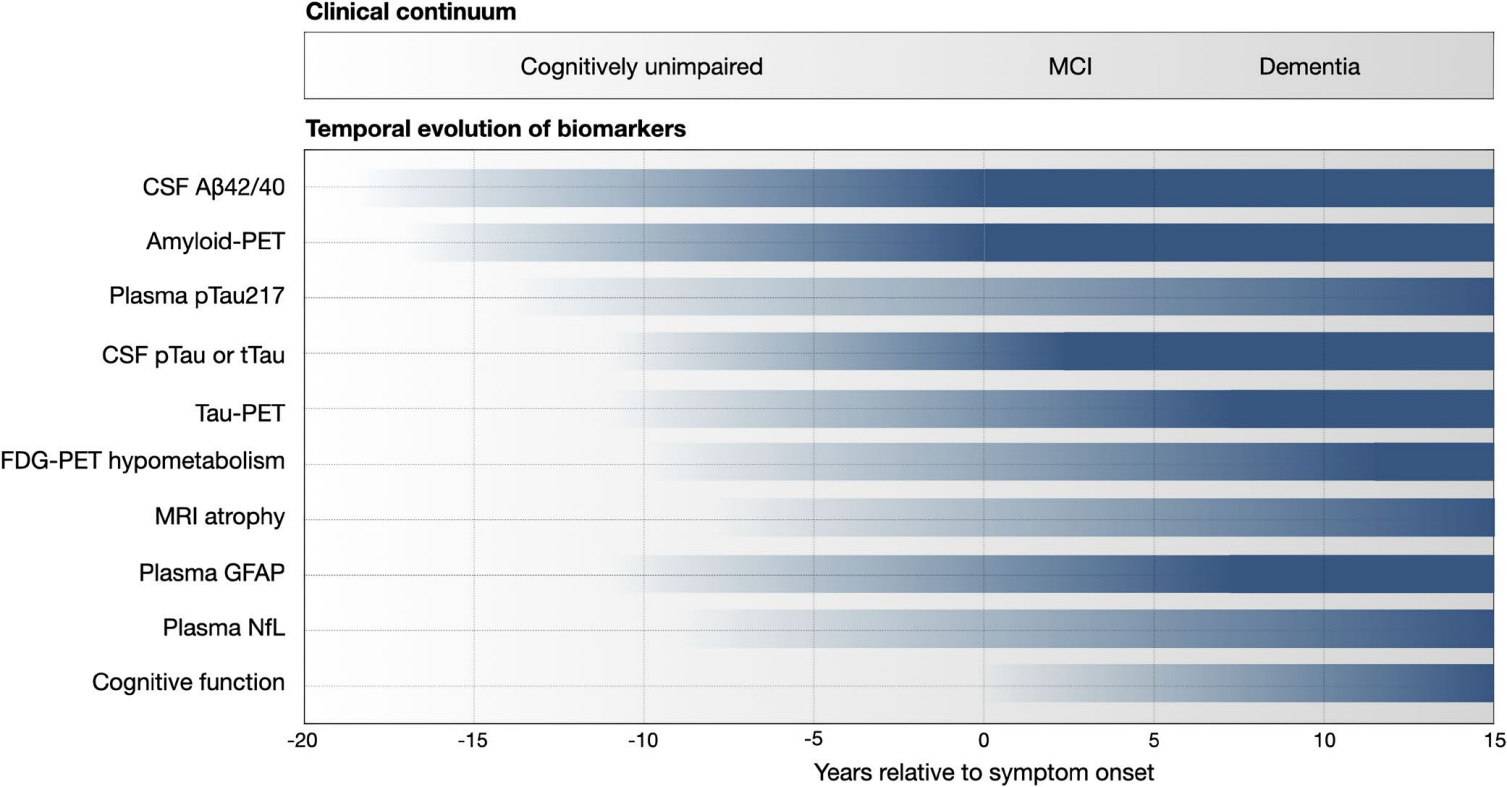
Clinical continuum and temporal evolution of biomarkers in Alzheimer's Disease. Biomarker changes are shown relative to the estimated time of symptom onset (year 0), across stages from cognitively unimpaired to mild cognitive impairment (MCI) and dementia. Darker colours indicate greater abnormality. Timelines are approximate and based on group‐level data from previous longitudinal studies and individual trajectories may vary [14].

AD is now understood as a multistage neurodegenerative disease, characterised by a long preclinical period during which pathology accumulates silently in the brain (Figure 1). Decades before the onset of dementia, a sequence of biological changes unfolds, including the accumulation of amyloid‐β (Aβ) protein, the aggregation of hyperphosphorylated tau protein (pTau) into neurofibrillary tangles, synaptic dysfunction, and progressive neuronal loss [14, 15]. While Aβ deposition is thought to initiate the cascade, tau pathology correlates more strongly with neuronal injury and clinical symptoms, typically observed early in the entorhinal cortex and hippocampus before spreading to associative cortices [16].

Reflecting the progressive nature of underlying pathology, the phenotype is conceptualised as a clinical continuum, from cognitively unimpaired individuals to MCI, and ultimately dementia. MCI represents a transitional stage, with measurable cognitive deficits that do not yet interfere with independence.

The 2024 research framework by the Alzheimer's Association workgroup defines AD biologically, based on amyloid pathology, irrespective of symptoms [5]. Cognitively unimpaired individuals with abnormal biomarkers are therefore considered to have “preclinical AD,” facilitating research and prevention trials. In contrast, the IWG requires both a compatible clinical syndrome and biomarker confirmation for an AD diagnosis [6]. Amyloid‐positive individuals without objective cognitive impairment are classified as “at risk,” reflecting uncertainty about progression and the risks of overmedicalisation. The IWG reserves pre‐symptomatic AD for individuals with a very high lifetime risk of progression (> 50%), such as autosomal dominant familial AD.

Importantly, Aβ positivity alone is an unreliable predictor of clinical progression. Although amyloid accumulation is a defining early event, many individuals with biomarker evidence of Aβ pathology remain clinically stable for years or progress more slowly than expected, probably due to brain resilience [5, 6]. Amyloid positivity also becomes increasingly common with age, even among cognitively normal individuals, rising from approximately 16% at age 60 to around one‐third (~33%) by age 80 [17]. Yet, dementia risk remains limited. For example, a 65‐year‐old man with positive amyloid biomarkers has an estimated lifetime risk of developing AD dementia of 21.9%, only moderately higher than his amyloid‐negative counterpart (12.9%) [18]. In another longitudinal study, fewer than 10% of cognitively normal, amyloid‐positive individuals progressed to MCI by four years [19].

Moreover, attributing cognitive symptoms solely to AD pathology can be misleading. Co‐pathologies, such as TDP‐43 pathology, cerebrovascular lesions, and α‐synuclein pathology, are common even in young‐onset AD and can significantly influence the clinical presentation and progression [5, 20, 21, 22]. Furthermore, up to 50%–75% of individuals with dementia with Lewy bodies (DLB) are also amyloid positive [23].

Clinically, AD most commonly presents with progressive episodic memory impairment. This typical or “amnestic” form is characterised by increasing forgetfulness, disorientation in time and place, and difficulty learning or recalling new information. Atypical presentations are less frequent and occur more often in younger individuals, where they may account for up to one‐third of cases [24]. These include language problems, as seen in the logopenic variant of primary progressive aphasia (lvPPA), where patients have difficulty retrieving words, producing sentences with frequent pauses [25]. In other patients, the first symptoms may involve visual processing, such as difficulty reading or judging distances, a presentation known as posterior cortical atrophy (PCA) [26]. Others may initially exhibit changes in executive function or behaviour, referred to as the dysexecutive or behavioural variant of AD [27].

Taken together, these findings support careful interpretation of biomarkers considering the clinical presentation, and vice versa. While biological definitions of AD enable early research and intervention, clinical judgement remains crucial to guide diagnosis and care. For routine clinical diagnosis of AD, we align with the IWG clinical‐biological construct, which requires both a compatible clinical syndrome and biomarker confirmation of AD pathology. The risk of overdiagnosis is particularly relevant in older individuals and in non‐amnestic syndromes, where the positive predictive value of amyloid biomarkers is lower.

## 3 Clinical Evaluation in the Diagnostic Work‐Up of Alzheimer's Disease

**FIGURE 2:**
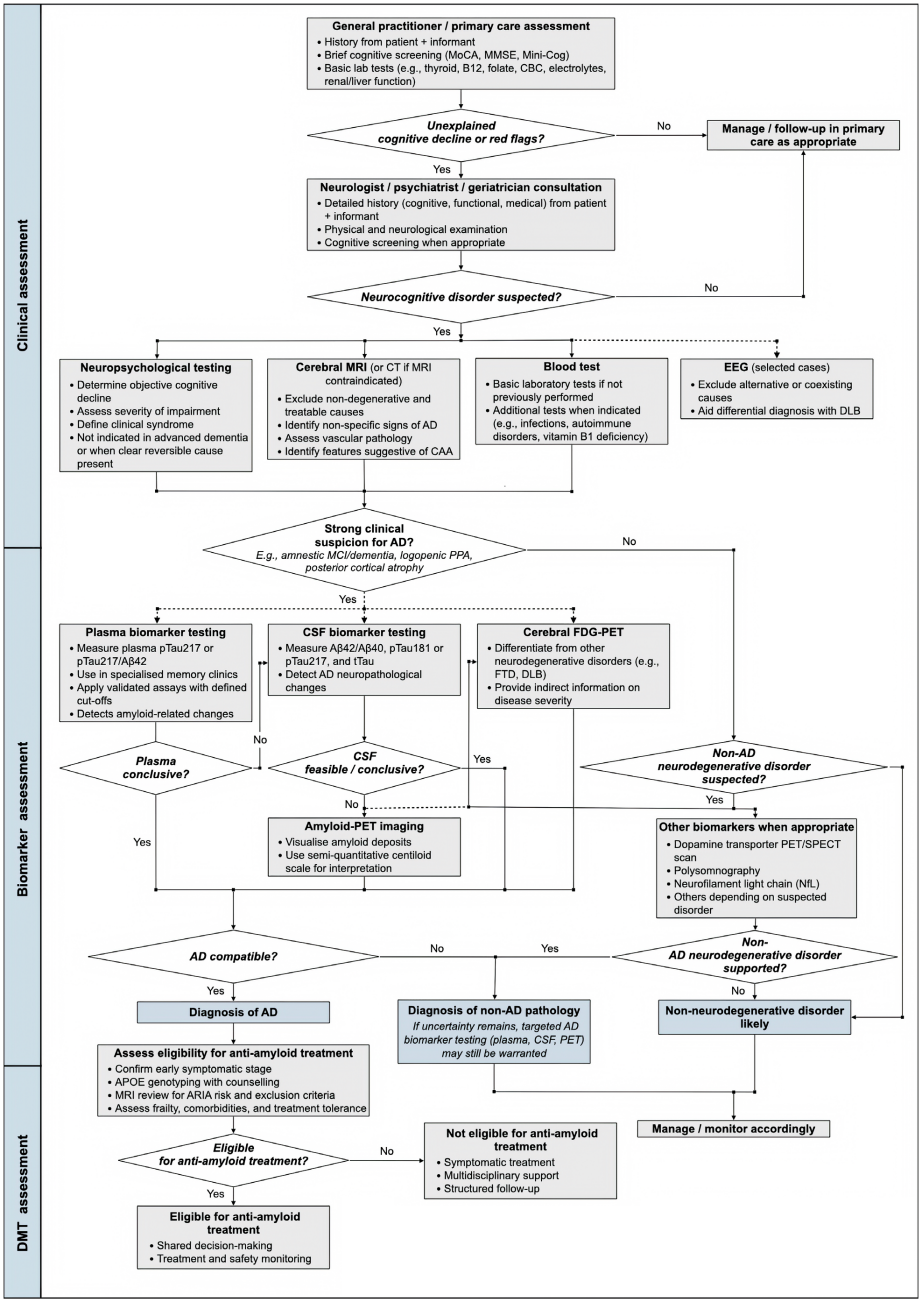
Stepwise diagnostic workflow for suspected Alzheimer's disease, with post‐diagnostic assessment of treatment eligibility. The workflow comprises three main clinical branches: (1) strong clinical suspicion for AD, leading to stepwise biomarker confirmation via plasma biomarkers, CSF analysis, and/or amyloid‐PET; (2) low clinical suspicion, leading to investigation of alternative neurodegenerative or non‐neurodegenerative diagnoses; and (3) post‐diagnostic assessment of eligibility for anti‐amyloid treatment. Solid lines indicate the recommended diagnostic sequence; dashed lines indicate optional or conditional pathways based on clinical judgement and local availability. AD = Alzheimer's disease, ARIA = amyloid‐related imaging abnormalities, CAA = cerebral amyloid angiopathy, DLB = dementia with Lewy bodies, FTD = frontotemporal dementia, LP = lumbar puncture, MCI = mild cognitive impairment, PCA = posterior cortical atrophy, PPA = primary progressive aphasia.

In line with international guidelines for the evaluation of neurocognitive disorders, we recommend a stepwise approach to determine both disease severity and the underlying clinical syndrome (Figure 2) [12, 28]. Such a structured process improves diagnostic accuracy and ensures appropriate referral and treatment planning in both primary and specialist care.

This process begins with a detailed history, including input from an informant, followed by physical examination, cognitive testing, neuroimaging, and laboratory screening. Input from a reliable informant is especially important, as individuals with cognitive decline may lack insight or provide an unreliable history. This collateral information often reveals additional changes in cognition, daily functioning, personality, or symptoms such as REM sleep behaviour disorder that are not reported or recognised by the patient.

Initial evaluation and cognitive screening may take place in primary care, while structured cognitive assessments and biomarker diagnostics are typically performed in specialist outpatient settings. Commonly used brief cognitive screening instruments include, among others, the MMSE, the MoCA, and the Mini‐Cog, each with specific strengths and limitations. The choice may depend on available time, patient population, and clinician familiarity [29]. A decent cognitive evaluation requires time and resources, including trained personnel such as neuropsychologists, specialist nurses, or physicians experienced in cognitive disorders, as well as validated tools. Blood tests help exclude systemic causes, such as thyroid dysfunction or vitamin deficiencies, and identify relevant comorbidities. Additional blood testing, such as infectious serology or autoimmune markers, may be indicated depending on the context.

Neuropsychological evaluation assesses core cognitive domains including memory, attention, language, executive functioning, visuospatial abilities, and social cognition. These results help determine the severity of impairment and support identification of a clinical syndrome. Formal testing is recommended in early symptomatic patients to improve diagnostic sensitivity and specificity, distinguish normal ageing from MCI and dementia, and aid in differentiating neurodegenerative from psychiatric conditions [30, 31]. It enables MCI subtyping, which may inform prognosis and therapeutic planning. The findings may also guide cognitive rehabilitation and practical decisions, such as driving ability or legal capacity [31].

Neuropsychological testing is less indicated in advanced dementia, where it is unlikely to alter management [32], although it may still aid objective monitoring. Formal testing may also be unreliable in patients with major language barriers, low literacy, acute psychiatric symptoms, or behavioural problems limiting cooperation. When reversible causes such as untreated obstructive sleep apnoea are suspected, it is advisable to treat these first and reassess cognition afterwards.

Neuropsychological assessment is resource‐intensive and may delay diagnosis. It adds value in many cases, but in some patients, if deficits are clearly documented by a memory specialist with validated tools, it may be reasonable to proceed directly with biomarker testing and defer formal assessment.

While standard criteria define MCI due to AD as objective impairment in one or more domains with preserved independence, and dementia as impairment in at least two domains with functional decline, these thresholds are not absolute as functional status is influenced by premorbid abilities, comorbidities, environmental support, and life complexity. Thus, the same cognitive profile may translate into different levels of disability across individuals. Recognising this variability is essential when evaluating disease severity and treatment eligibility [31].

Subtyping of MCI is clinically relevant. Amnestic single‐domain MCI carries the highest likelihood of underlying AD pathology and progression to dementia. In contrast, multi‐domain or non‐amnestic MCI often suggests alternative or mixed aetiologies. In a clinic‐based study, amyloid‐PET positivity was observed in ~65% of amnestic MCI cases compared to 25% of non‐amnestic cases [33]. Amyloid positivity increases with age, even among cognitively normal individuals, and must be considered when interpreting biomarker results. Thus, amyloid positivity in non‐amnestic MCI may reflect atypical AD, asymptomatic AD co‐pathology, or another amyloid‐related process such as cerebral amyloid angiopathy (CAA).

The clinical phenotype provides additional diagnostic guidance. Certain syndromes, such as the amnestic syndrome, lvPPA, or PCA, are closely associated with Alzheimer's pathology. In contrast, other phenotypes, including prominent behavioural changes, early dysexecutive symptoms, or parkinsonism with hallucinations, are more suggestive of frontotemporal lobar degeneration or DLB. Similarly, features such as vertical gaze palsy, early postural instability, or axial rigidity may indicate progressive supranuclear palsy (PSP), which is typically not associated with AD pathology.

Structural neuroimaging, preferably MRI without gadolinium or CT when MRI is contraindicated, is a key component of the diagnostic assessment of cognitive disorders [34]. It serves to exclude structural and potentially treatable causes such as meningioma, subdural hematoma, or normal pressure hydrocephalus (NPH). MRI is also important for assessing vascular pathology, including white matter hyperintensities, lacunes, infarcts, and microbleeds, which may contribute to cognitive impairment. In addition, MRI is essential for treatment planning with anti‐amyloid therapies, as it can detect markers of CAA, which are associated with a higher risk of ARIA, and monitor ARIA. Baseline and follow‐up scans are therefore required to ensure eligibility and safety during therapy [35, 36]. Patterns of cortical atrophy may also support the differential diagnosis, with their role as biomarkers of AD discussed in Section 4.1. At minimum, a baseline MRI protocol for the diagnostic assessment of cognitive disorders should include a 3D T1‐weighted sequence (for volumetric and atrophy assessment), a FLAIR sequence (for white matter hyperintensities and vascular pathology), a DWI sequence (to detect acute infarcts and assess for rapidly progressive disorders such as CJD), and a susceptibility‐sensitive sequence such as T2‐weighted GRE or SWI (for detection of microbleeds and superficial siderosis, and for ARIA safety screening before anti‐amyloid therapy) [37].

Routine electroencephalography (EEG) has a limited role in the diagnostic work‐up of AD and is not included in the biomarker‐based diagnostic pathway outlined in this consensus [38]. Nonetheless, EEG can be valuable in selected patients to assess alternative or coexisting causes of cognitive decline, such as epilepsy, toxic‐metabolic encephalopathy, or rapidly progressive dementias like Creutzfeldt‐Jakob disease, and can also support a diagnosis of DLB [39].

## 4 Biomarkers for Alzheimer's Disease: Use and Limitations

Biomarkers are objectively measured biological indicators that reflect normal physiology, pathogenic processes, or responses to therapeutic interventions [40]. In AD, biomarkers play an important role in diagnosis and staging, particularly in identifying key pathological features, such as amyloid plaques, tau tangles, and neurodegeneration.

The clinical value of amyloid biomarkers depends not only on their analytical accuracy but also on the prevalence of amyloid positivity in the tested population (= the pretest probability). Even highly sensitive and specific tests may yield considerable false positives in populations with low pretest probability of AD pathology, such as cognitively unimpaired individuals or patients with non‐amnestic syndromes [41, 42].

In clinical practice, biomarker testing should be reserved for individuals with objective cognitive decline, typically when AD is part of the differential diagnosis (Figure 2) [43]. Testing in cognitively unimpaired individuals, including those with only subjective complaints, is currently clinically not recommended given the limited predictive value, risk of misinterpretation, and lack of therapeutic consequences. Further, as noted above, biomarker results must always be interpreted in clinical context, as comorbidities and mixed pathologies are common.

### 4.1 Non‐Specific Biomarkers

Structural MRI is part of the general diagnostic work‐up but can also serve as an indirect AD biomarker by detecting patterns of neurodegeneration. Visual assessment of regional atrophy is widely used, with scales such as medial temporal and posterior atrophy scales that are simple, inexpensive, and of moderate diagnostic value in more advanced disease [44]. However, their sensitivity and specificity are lower in early stages (e.g., MCI) and in younger or atypical cases, and performance is further limited by variability in interpretation and thresholds [45, 46]. Medial temporal atrophy is not specific for AD, particularly in older individuals, as it may also reflect hippocampal sclerosis or limbic‐predominant age‐related TDP‐43 encephalopathy (LATE) [47]. Automated volumetric analysis may address some of these limitations and improve accuracy in routine care, but use remains constrained by software variability, cost, lack of standardisation, and incomplete clinical validation [48, 49].

FDG‐PET is a functional imaging technique reflecting neuronal activity and is more sensitive than MRI for detecting early neurodegenerative disease. A characteristic pattern of reduced glucose metabolism in the posterior cingulate, precuneus, and lateral temporoparietal regions has high specificity for classical amnestic AD [50]. FDG‐PET also helps differentiate AD from frontotemporal dementia, LATE, or DLB, and distinguishes AD subtypes through distinct hypometabolic patterns [50]. Although not pathology‐specific like amyloid‐ or tau‐PET, it is a valuable adjunct when clinical features or fluid biomarkers remain uncertain, including intermediate or discordant CSF or blood‐based results. It can also predict short‐term conversion from MCI to dementia due to AD, where it has been shown to outperform amyloid‐PET [51], and it provides complementary information on disease severity, as clinical status closely correlates with metabolic patterns [52]. Computer‐assisted techniques such as Z‐map renderings can further improve diagnostic accuracy and provide quantitative measures of pathological findings [53].

### 4.2 Specific Alzheimer Biomarkers

CSF and molecular PET biomarkers have transformed AD diagnostics, enabling reliable in vivo detection of pathology. In many European countries, CSF testing is the most widely used and accessible approach. Amyloid plaques are inferred from reduced β‐amyloid 1–42 (Aβ42) levels in CSF, as this peptide is sequestered in insoluble aggregates [54, 55]. Because Aβ42 levels vary with individual production and pre‐analytical factors, the Aβ42/Aβ40 ratio is recommended, as it is more accurate than measuring Aβ42 alone [56, 57]. In autopsy‐confirmed cohorts, such ratios achieve sensitivities and specificities around 90%, supporting their role as highly reliable indicators of amyloid pathology [58].

Classically, the CSF marker for amyloidosis is interpreted in conjunction with levels of tau species in CSF. Elevated pTau181 or pTau217 and total tau (tTau) reflect downstream tau phosphorylation and neuronal injury, respectively. When both amyloid and tau markers are abnormal, the specificity for AD is very high, particularly in patients with MCI or mild dementia [59].

CSF results are not always definitive. Some patients show abnormal amyloid markers while tau remains normal or borderline. An isolated decrease in the Aβ42/Aβ40 ratio has relatively low specificity and may lead to misclassification as AD. This A‐positive/T‐negative profile may reflect early biological AD in which tau changes are not yet detectable, but might also occur in DLB, CAA, and NPH [60, 61]. Still, in early disease stages, a reduced Aβ42/Aβ40 ratio can be biologically meaningful when supported by compatible clinical features [62]. In such discordant situations, we suggest (1) re‐evaluating whether the clinical presentation remains most consistent with AD or instead suggests an alternative diagnosis, (2) considering FDG‐PET to assess for an AD‐typical hypometabolic pattern, (3) if uncertainty persists, considering amyloid‐PET, and (4) arranging clinical and, where appropriate, biomarker follow‐up [63]. Finally, lumbar puncture may be perceived negatively or refused by patients and families, limiting use and requiring careful discussion.

Amyloid‐PET is a non‐invasive alternative to CSF to confirm amyloid status by visualising cortical fibrillar deposits. Although CSF and amyloid‐PET can be used interchangeably in most indications, they provide different information: PET imaging shows aggregates and spatial distribution, while fluid biomarkers reflect protein dynamics at a given time. In current clinical practice in Belgium, amyloid‐PET is mainly reserved for patients in whom lumbar puncture is not feasible or carries risk, such as spinal abnormalities, anticoagulant use where interruption is unsafe, or refusal due to anxiety [64]. It may also be considered when CSF results are inconclusive despite strong clinical suspicion of AD. Amyloid‐PET can also guide treatment discontinuation in donanemab. In the phase 3 TRAILBLAZER‐ALZ study, therapy was stopped once amyloid clearance was demonstrated on repeat amyloid‐PET, though feasibility in practice remains uncertain, with limited reimbursement for amyloid scans as the main issue. Besides visual assessment, semi‐quantitative analysis using the centiloid scale is recommended, as endorsed by the EMA and FDA. Multiple commercially available [18F]‐labelled tracers are in clinical use.

Tau‐PET imaging is a more recent development that enables in vivo visualisation of neurofibrillary tangles [65]. Tauvid ([^18^F]flortaucipir), the first tau‐PET tracer, was approved by the EMA for clinical use in Europe in August 2024, but it is currently not reimbursed in Belgium. Second‐generation tau‐PET tracers, with improved binding characteristics such as [^18^F]MK6240 and [^18^F]PI2620, are available in Belgium through academic studies and industry‐sponsored clinical trials. Tau‐PET has been shown to be strongly associated with cognitive impairment [66], and shows high specificity for AD‐type pathology compared with non‐AD neurodegenerative pathologies [67]. Tau‐PET may help to confirm AD pathology in atypical presentations such as PCA or lvPPA, and in A+T‐CSF cases, it may help determine whether AD pathology is the primary driver of the cognitive disorder, or whether alternative or contributing aetiologies should be considered [5]. Unlike amyloid‐PET, which often shows positivity in cognitively unimpaired older individuals, neocortical tau pathology detected by tau‐PET is much less prevalent in this group, ranging from 3% at age 60 to 19% at age 90. When present, it has greater prognostic relevance: among tau‐positive individuals, 57% develop MCI or AD dementia within 6 years [68, 69].

### 4.3 Blood‐Based Biomarkers

Blood‐based biomarkers are a rapidly emerging diagnostic tool for AD, providing a less invasive, scalable, and cost‐effective alternative to CSF testing and PET imaging [70].

Among these, plasma pTau217 shows the strongest evidence and is likely to become a first‐line triage tool in specialised memory clinics [71, 72]. This marker is thought to reflect amyloid‐induced tau phosphorylation and shows high concordance with CSF pTau217 [73]. Plasma pTau217 has high diagnostic accuracy across the AD clinical continuum, with pooled sensitivity and specificity near 90%, and area under the curve (AUC) values above 90% in well‐characterised cohorts validated against CSF or amyloid‐PET [71]. It also helps distinguish AD from other neurodegenerative disorders [74], and may detect pathological changes in cognitively unimpaired individuals at risk for AD, even before tau‐PET becomes abnormal [75]. It should be noted that much of this evidence derives from selected research cohorts, and that real‐world diagnostic performance may be lower. As noted above, diagnostic accuracy depends on pre‐test probability, and inter‐assay and inter‐laboratory variability remains a relevant limitation that must be addressed before routine clinical deployment.

The selection of appropriate thresholds must be tailored to the intended clinical use. In a screening or triage context, a lower threshold prioritising high sensitivity is needed to reliably rule out amyloid pathology and minimise missed cases. In specialist memory clinics, when the aim is diagnostic confirmation, higher thresholds with greater specificity are more appropriate to minimise false positives and support treatment decisions [41].

Plasma pTau181 was the first phospho‐tau variant widely studied in blood and shows utility as an AD biomarker, but pTau217 has consistently shown higher accuracy for detecting abnormal Aβ status [73].

For plasma pTau217 interpretation in specialist memory clinics, a two‐cut‐off approach may be advisable [41, 70, 76]. Levels above the higher cut‐off indicate amyloid positivity, those below the lower cut‐off indicate amyloid negativity (consistent with the amyloid, and subsequent tau cascade), while intermediate results should prompt confirmatory CSF or amyloid‐PET testing [77]. When sensitivity and specificity cut‐offs are set at 95%, studies in secondary care cohorts report that 12%–17% of individuals fall into the intermediate range [78].

By contrast, plasma Aβ42 and Aβ40 levels, typically used as the Aβ42/Aβ40 ratio, show more modest diagnostic performance. Although a lower Aβ42/Aβ40 ratio correlates with amyloid‐PET positivity, its utility is limited by small group differences, pre‐analytical variability, and assay‐dependent effects [55, 79]. Accuracy improves with high‐precision platforms such as immunoprecipitation mass spectrometry, but these are not yet widely accessible, and standardisation remains a challenge.

Recent studies suggest that combining plasma Aβ42 with pTau217, as the pTau217/Aβ42 ratio, may enhance accuracy and reduce the intermediate zone [80, 81]. Further research in real‐world settings is needed to determine whether the added value justifies the extra cost of plasma Aβ42 analysis. In 2025, the FDA approved the plasma pTau217/Aβ42 ratio as an aid for the early detection of amyloid plaques in adults aged ≥ 55 years with cognitive symptoms.

Neurofilament light chain (NfL) and glial fibrillary acidic protein (GFAP) are additional plasma biomarkers of neurodegeneration and astroglial activation, respectively. NfL may help distinguish neurodegenerative from non‐neurodegenerative causes of cognitive symptoms but lacks specificity [82]. GFAP shows a stronger association with AD pathology [83], although clinical thresholds remain to be established. While less accurate than pTau217, both may provide complementary diagnostic information.

Finally, certain systemic conditions, such as impaired kidney function, may influence plasma biomarker levels and potentially affect test interpretation [84]. Although the impact of such cofactors appears to be modest, they underscore the importance of interpreting blood‐based biomarkers within the overall clinical and biological profile [85]. Although most validation studies have been conducted in predominantly European ancestry cohorts, emerging evidence suggests that diagnostic accuracy may be maintained across diverse populations [86], but this needs further confirmation.

Plasma biomarkers are moving rapidly from research into clinical practice but are not yet reimbursed in Belgium. In May 2026, both Fujirebio (Lumipulse G pTau 217 Plasma) and Roche (Elecsys pTau217) received CE‐IVD certification, the European regulatory approval for clinical diagnostic use, marking an important step towards routine clinical implementation. The Roche Elecsys pTau181 had received the same certification in July 2025. Nevertheless, plasma pTau217, and potentially the pTau217/Aβ42 ratio, show strong promise as minimally invasive, scalable tools to guide referrals for confirmatory CSF or PET testing. A cautious approach is warranted: initial use should be limited to specialist memory clinics, where the pretest probability of amyloid pathology is higher, and results can be interpreted in a multidisciplinary context. Safe implementation benefits from CE‐IVD‐certified platforms now available. Ongoing standardisation of cut‐offs across centres remains essential to minimise diagnostic errors. Confirmatory CSF or PET remains essential in indeterminate cases. Use in primary care or as a general screening tool is premature and risks misdiagnosis or unnecessary downstream testing. Although recently approved for primary care use in the US, a clinical practice guideline restricts blood‐based biomarker recommendations to specialised care settings, citing lower pre‐test probability, limited non‐specialist experience in interpreting biomarker results, and barriers to integration in primary care workflows [87]. Whether and how blood‐based biomarkers can be reliably deployed in primary care or geriatric outpatient settings to prioritise referrals requires further implementation research before broader adoption can be recommended. This challenge is not unique to AD; similar concerns were raised with PSA testing for prostate cancer, underscoring the need to apply biomarkers within the clinical context [88].

Beyond AD biomarkers, other markers may help exclude alternative or contributing causes of cognitive decline, as shown in Figure 2. Table 3 compares available biomarker modalities, and their recommended role is summarised in Box 1.

**TABLE 3:** Overview of current and emerging biomarker modalities for Alzheimer's disease.

Biomarker	Target	Strengths	Limitations	Recommendations	Regulatory approval (Europe)	Reimbursement (Belgium, early 2026)
Structural MRI	Structural changes, atrophy patterns, vascular pathology	Widely available; supports DD; essential for ARIA safety screening	Low sensitivity in early stages; inter‐rater variability	Core part of dementia work‐up	Yes	Reimbursed
FDG‐PET	Regional glucose metabolism	Sensitive to early neurodegenerative change; characteristic patterns support DD	Indirect aetiological marker; higher cost	Atypical or inconclusive cases; may inform disease stage/severity	Yes	Reimbursed under specific conditions
CSF Aβ42/Aβ40 + pTau (181/217) + tTau	Amyloid & tau pathology	High diagnostic accuracy; available in reference labs	Invasive LP; pre‐analytical variability	First‐line AD biomarker confirmation in memory clinics	Yes	Not reimbursed
Amyloid‐PET	Amyloid plaques	High diagnostic accuracy; quantifiable; alternative to LP	Amyloid‐only marker; cost; limited access; logistics	Second‐line if LP not feasible or inconclusive	Yes	Limited reimbursement
Tau‐PET	Neurofibrillary tangles	Correlates with symptoms/stage; high specificity for AD	Limited access; validation ongoing	Complex differential diagnosis; potential severity/progression marker	Yes	Not reimbursed
Plasma pTau217	Amyloid‐induced tau phosphorylation	Minimally invasive; high accuracy; early triage potential	Assay dependent cut‐offs; comorbidity may influence interpretation; intermediate zone	Potential use for triage in memory clinics; assess indeterminate results with CSF or amyloid‐PET	Yes	Not reimbursed
Plasma pTau181	Tau phosphorylation	Minimally invasive; useful alternative	Less accurate than pTau217	Alternative if pTau217 not available	Yes	Not reimbursed
Plasma Aβ42/Aβ40	Amyloid metabolism	Minimally invasive, amyloid screening potential	Small group differences; pre‐analytical variability	May improve pTau interpretation; not for stand‐alone use	No	Not reimbursed
Plasma NfL	Axonal injury	Marker of neurodegeneration; potential prognostic information	Not AD‐specific; influenced by age and comorbidity	Potential severity/progression marker, not diagnosis	Yes	Not reimbursed
Plasma GFAP	Astroglial activation	Linked to amyloid/tau; early rise possible	Low specificity; no validated cut‐offs	Complementary; mainly research	No	Not reimbursed

*Note:* Regulatory approval refers to EMA marketing authorisation (imaging tracers), CE marking under MDR (MRI), or CE‐IVD certification under IVDR (CSF and blood‐based assays). For PET and fluid biomarkers, regulatory status is tracer‐ assay‐ or indication‐dependent. DD = differential diagnosis. LP = lumbar puncture.

## 5 Genetic Considerations in Alzheimer's Disease

Age is the strongest risk factor for AD, with prevalence rising exponentially after 65. Genetic factors also play a crucial role, with heritability estimated at 60%–70% [89]. The remaining risk is generally attributed to modifiable or environmental factors, including education, hypertension, physical inactivity, hearing impairment, depression, and social isolation [90].

Most genetic risk is polygenic, with common variants at > 75 loci each conferring small effects [91].

A notable exception is the *APOE* gene, which exists in three common allelic forms: ε2, ε3, and ε4. The ε3 allele is most frequent. In Belgian controls, estimated frequencies are approximately 79% for ε3, 13% for ε4, and 8% for ε2 [92, 93]. Correspondingly, ~20% are ε3/ε4 heterozygotes and 1%–2% ε4/ε4 homozygotes. The ε4 allele is the strongest genetic risk factor for late‐onset AD: heterozygotes have a 3–4‐fold, and homozygotes a 10–15‐fold increased risk compared to ε3/ε3 carriers [93, 94]. In contrast, the ε2 allele reduces risk.

Lifetime risk estimates vary by age and sex, but population‐based data suggest that ε4/ε4 homozygotes have up to a 65% risk of developing dementia by age 85, ε3/ε4 individuals 25%–35%, and ε3/ε3 carriers ~10% risk [94]. Importantly, *APOE* ε4 is neither necessary nor sufficient to cause AD: a substantial proportion of ε4 carriers remain unaffected, and non‐carriers can also develop the disease.

Until recently, *APOE* genotyping was generally discouraged in clinical practice because of its limited predictive value and potential psychological harm [95]. With anti‐amyloid therapies, *APOE* ε4 status is now relevant for ARIA risk. US guidance advises genotyping candidates to inform risk discussions and monitoring [36, 96], while EMA requires genotyping before treatment initiation and excludes ε4/ε4 homozygotes. *APOE* testing remains contraindicated in asymptomatic individuals, since no therapeutic action follows a positive result.

Monogenic AD is extremely rare, accounting for < 1% of patients, and is usually caused by autosomal dominant pathogenic mutations in *APP*, *PSEN1*, or *PSEN2* [97]. It typically presents before age 65, often in the 40s–50s. Rare variants in *TREM2*, *ABCA7*, and *SORL1* also confer moderate‐to‐high, but incomplete, risk [98].

*APOE* genotyping should generally be restricted to patients considered for anti‐amyloid therapy. Genotype‐specific ARIA incidence rates and management recommendations are detailed in published appropriate use recommendations [36, 96] and should inform pre‐treatment counselling. In these patients, we recommend that pre‐ and post‐test counselling be provided by a genetics professional or a relevant specialist (neurologist, psychiatrist, or geriatrician), as also highlighted in Box 1. Counselling should prepare patients and families for medical implications (treatment eligibility, ARIA risk), psychological impact, consequences for relatives, and potential effects on insurance or long‐term care planning [99]. Genetic testing for monogenic AD should generally be considered in symptomatic early‐onset patients or those with an early‐onset autosomal dominant family history, and can be initiated by the memory clinic specialist. Predictive testing may be offered to asymptomatic relatives with a known mutation in the family but must be coordinated through a clinical genetics service, as required by Belgian regulations.

Genetic insights continue to advance our understanding of AD pathogenesis and may increasingly support risk stratification, prognostic refinement, and personalised therapies. As with all biomarker and genetic testing, procedures must comply with GDPR and national data protection legislation, with consent covering data storage, sharing, and potential future use. Ethical considerations include appropriate counselling, informed consent, and adequate support.

## 6 Care Organisation and System Preparedness in Belgium

The implementation of DMTs for AD requires both diagnostic precision and a healthcare system capable of meeting increased demands on capacity, coordination, and equity. These therapies, together with the need for biomarker‐based diagnostics, will place significant strain on existing care pathways. Although anti‐amyloid treatments have been approved in Europe, they are not yet reimbursed in Belgium.

In Belgium, dementia care remains fragmented across regions, providers, and levels of care. This lack of structural integration creates barriers to early and timely diagnosis, treatment initiation, and long‐term follow up, and risks widening disparities in access to specialised care. Addressing these gaps is essential to ensure safe and timely access to new therapies for all eligible patients.

### 6.1 Current Alzheimer's Disease Care Organisation in Belgium

Belgian citizens generally have easy access to their general practitioner (GP), with 83.3% having a registered medical file in a GP practice (referred to in Belgium as the global medical dossier, a system of coordinated primary care; Flanders 87.8%, Wallonia 79.6%, Brussels 67.8%; www.gezondbelgie.be, consulted on 04/08/2025). GPs and other primary care providers can detect early signs of cognitive dysfunction and refer to secondary care for further diagnostics, although patients can also directly access specialists. Validated guidelines exist for diagnosis and multidisciplinary collaboration in primary care, but implementation remains inadequate [100].

The organisation of memory clinics is a key structural element in dementia care. In the late 2000s, 12 clinics were officially recognised and continue to receive federal funding for reimbursed cognitive rehabilitation in early dementia, provided patients are expected to remain at home for at least 12 months. These programmes aim to help patients cope with limitations and maintain independence, while supporting caregivers through education and practical advice. However, no new clinics have been recognised since 2010, and the criteria for recognition have not been revised or extended to cover diagnosis and treatment. As a result, the current care landscape includes both memory clinics with a recognised rehabilitation programme and those without, leading to considerable variation in distribution and capacity across regions.

The Flanders Centre of Expertise on Dementia (Expertisecentrum Dementie Vlaanderen vzw) and its regional centres are officially recognised partners of the Flemish government. They mainly support community‐based care through post‐diagnostic guidance, training, education, public awareness, and by bridging clinical practice and research. Together, they promote person‐centred and integrated dementia care according to the Flemish reference framework [101]. Since 2024, Flanders has trained “Dementia Reference Physicians” to support healthcare professionals in primary care and, where necessary, in secondary care on dementia‐related issues. These physicians work in collaboration with the Flanders Centre of Expertise on Dementia and operate within the 60 primary care zones [102].

Other key partners include home care services, local social welfare offices, and patient organisations such as Alzheimer Liga and Alzheimer Belgique. These groups provide information, advocacy, and peer support, helping people live at home as long as possible. For young‐onset dementia, buddy services and specialised programmes are available in 23 nursing homes.

### 6.2 Challenges in Preparing the Alzheimer's Disease Care System for Disease‐Modifying Therapies

The introduction of DMTs requires a structured and clearly defined diagnostic pathway. Eligible early symptomatic AD patients must be identified through a multistep process, starting in primary care and continuing in specialised, multidisciplinary memory clinics. Access to and correct interpretation of biomarker‐based tests (blood, CSF, amyloid‐PET) is critical. Uneven availability and familiarity with these tests across centres risk differences in diagnostic accuracy and treatment eligibility. Access to MRI and PET is already limited, as scanner numbers are regulated. Streamlined workflows and dedicated capacity for mandatory imaging will be essential to avoid delays in treatment initiation.

Professional capacity is also insufficient, particularly in cognitive neurology, geriatrics, and neuropsychology, if early detection and regular monitoring become routine. Repeated intravenous infusions every 2–4 weeks will further increase demand for day‐hospital facilities and nursing staff. Multidisciplinary collaboration is essential to ensure appropriate use of biomarker‐based diagnostics and DMTs. In older patients, in whom AD is most prevalent, close collaboration between geriatrics and neurology is particularly important, with age alone not serving as an exclusion criterion. A Comprehensive Geriatric Assessment (CGA) can support personalised decision‐making by identifying those likely to benefit from DMTs versus those for whom supportive care is more appropriate, taking into account frailty, comorbidities, and tolerance of treatment and monitoring requirements [35]. Continuing and advanced training programmes for all healthcare professionals involved in the AD care pathway will be essential to address this challenge.

Reimbursement of diagnostic services remains inconsistent. GP and specialist consultations, blood tests, and brain imaging (e.g., MRI) are partly reimbursed, with co‐payments. Neuropsychological testing is only partially reimbursed once during an initial ambulatory evaluation, requiring a GP referral. Repeat testing is reimbursed once in patients under 75 but more frequently in those over 75 when performed in a geriatric day hospital. Outside this setting, patients often face out‐of‐pocket costs of €150–250 or more. CSF biomarker testing (~€100 or more) and amyloid‐PET imaging (tracer ~€1300, ~€60 reimbursed; scan partly covered under specific conditions) remain largely out‐of‐pocket. Blood‐based biomarkers, although FDA‐approved in the US, are not yet reimbursed in Belgium. Screening for monogenetic causes is reimbursed, as is *APOE* genotyping in a diagnostic context. The absence of reimbursement for biomarker testing creates inequities in access, as only patients able to pay out‐of‐pocket can obtain timely confirmation of the diagnosis. This barrier delays treatment initiation, prolongs diagnostic trajectories through unnecessary additional investigations, and reduces the overall efficiency of the healthcare system. Across Western Europe, reimbursement of CSF and amyloid‐PET are heterogeneous: CSF testing is reimbursed in some countries (e.g., France, Germany, UK), while amyloid‐PET is reimbursed in Sweden, and only in restricted contexts in France or via private insurance in Germany.

At the national level, Belgium currently lacks an up‐to‐date dementia strategy. The first Flemish dementia plan (2016–2020) emphasised public awareness and innovation, while the current plan (2021–2025) focuses on prevention, autonomy, and social participation. As ~70% of people with dementia live at home, the plan highlights the need to strengthen community‐based support systems. In this context, DMTs may help maintain independence longer and may delay admission to residential care, although this remains to be proven.

### 6.3 Recommendations

To translate biomarker‐based recommendations into routine practice (see Box 1), they must be embedded in an adequately prepared healthcare system. Several system‐level and policy adaptations are required to ensure safe, timely, and equitable implementation.

Access to diagnostic services, including CSF, blood‐based biomarkers, and amyloid‐PET, should be improved. Reimbursement structures for these tests and for neuropsychological evaluation require urgent revision to ensure appropriate assessment of eligible patients. The geographic distribution of memory clinics should be expanded and optimised through updated recognition criteria that also cover non‐pharmacological management. Targeted training programmes are needed to address workforce shortages in cognitive neurology, geriatrics, neuropsychology, and nursing. Finally, a coordinated national dementia policy is essential for equitable access, consistent standards, and sustainable implementation of DMTs across Belgium.

Beyond early detection and treatment, sustainable care also requires long‐term follow‐up of patients, families and caregivers by specialised teams. Many AD patients are likely not eligible for anti‐amyloid therapies in Europe, whether due to advanced disease stage, *APOE* ε4 homozygosity, safety contraindications, or significant comorbidities and frailty [103]. These patients equally require structured follow‐up, symptomatic treatment, and multidisciplinary support. Close collaboration between neurology, geriatrics, general practitioners and allied professionals, within integrated memory clinics, geriatric day hospitals and community‐based services, will be important to ensure continuity of care. With growing population diversity, culturally appropriate approaches in assessment, counselling and care are needed to support inclusive diagnostic and treatment strategies.

At the same time, innovation should be actively integrated: blood‐based biomarkers are likely to play an increasingly important role in care pathways, not only for diagnosis but potentially also for longitudinal monitoring, disease staging, and treatment response assessment. Plasma pTau217 is currently the most advanced candidate, but other markers may provide complementary value. Similarly, the role of amyloid‐ or tau‐PET may expand, for example in guiding treatment decisions. Broader clinical use will require clear indications, robust validation, and appropriate reimbursement.

While some recommendations in this consensus statement are specific to the Belgian healthcare system, particularly those related to reimbursement policies, regional care organisation and current biomarker availability, most principles are broadly applicable. They can be adapted to local resources, infrastructure and regulatory frameworks to support DMTs implementation in AD. In particular, the stepwise diagnostic approach, biomarker interpretation framework, and the emphasis on multidisciplinary collaboration and system readiness are relevant to any healthcare system preparing for DMTs.

This consensus has several strengths, including its broad multidisciplinary basis integrating expertise across the AD care pathway, its integration of diagnostic, ethical, and system‐level considerations into a single practical framework spanning the full diagnostic trajectory, and its practical focus on actionable recommendations for clinicians and policymakers. Limitations include the absence of a formal Delphi process or systematic grading of evidence, the rapidly evolving evidence base for blood‐based biomarkers and DMTs, and the lack of formal cost‐effectiveness modelling.

## 7 Conclusion

In summary, DMTs for AD require both accurate biomarker confirmation and system readiness. A stepwise diagnostic approach, embedded in well‐prepared memory services and supported by reimbursement reform, workforce training, and national policy coordination, is essential to ensure safe and equitable implementation. Blood‐based biomarkers will expand future diagnostic capacity, and their clinical use should be guided by robust validation. Ethical considerations and structured patient counselling should remain integral to all diagnostic pathways.

## Author Contributions

**Tim Van Langenhove:** conceptualization, writing – original draft, writing – review and editing, visualization, project administration. **Sara Van Mossevelde:** conceptualization, writing – original draft, writing – review and editing, visualization. **Marijke Miatton:** writing – original draft, writing – review and editing. **Rose Bruffaerts:** writing – review and editing. **Evert Thiery:** writing – review and editing. **Anne Sieben:** writing – review and editing. **Kurt Segers:** writing – review and editing. **Jan De Lepeleire:** writing – review and editing. **Jean Christophe Bier:** writing – review and editing. **Mirko Petrovic:** writing – review and editing. **Haroun Jedidi:** writing – review and editing. **Christian Gilles:** writing – review and editing. **Jurn Verschraegen:** writing – review and editing. **Eric Mormont:** writing – review and editing. **Manfredi Ventura:** writing – review and editing. **Donatienne Van Weehaeghe:** writing – review and editing. **Gaëtane Picard:** writing – review and editing. **Mélanie Strauss:** writing – review and editing. **Eric Salmon:** writing – review and editing. **Gert Cypers:** writing – review and editing. **Sebastiaan Engelborghs:** conceptualization, writing – review and editing, writing – original draft, supervision. **Peter De Deyn:** writing – review and editing. **Jan Versijpt:** writing – review and editing. **Olivier Deryck:** conceptualization, writing – original draft, writing – review and editing, supervision. **Rik Vandenberghe:** writing – review and editing. **Bernard Hanseeuw:** writing – review and editing, conceptualization, writing – original draft, supervision. **Joris Vlaemynck:** writing – review and editing. **Katrin Gillis:** writing – review and editing. **Maria Bjerke:** writing – review and editing. **Ingo Beyer:** writing – review and editing. **Aline Delva:** writing – review and editing.

## Conflicts of Interest

Tim Van Langenhove has received consulting fees from Eisai (paid to institution), and Eli Lilly (paid to institution). Sara Van Mossevelde has received consulting fees from Fujirebio (paid to institution), and Eisai (paid to institution). Marijke Miatton has received consulting fees from Eisai (paid to institution). Jan De Lepeleire has received consultancy fees from Eisai and Biogen. Rose Bruffaerts has received consulting fees from Eisai (paid to institution), and Eli Lilly (paid to institution). Jean Christophe Bier was supported by the Fonds Erasme pour la recherche médicale (‘Remember’ Convention). Donatienne Van Weehaeghe has received research support from General Electric, consulting fees from Eli Lilly, and reader honoraria from Life Molecular Imaging (paid to institution). Mélanie Strauss has received consulting fees from Eisai (paid to institution), and was supported by the Fonds Erasme pour la recherche médicale (‘Remember’ Convention) and the Fonds pour le Recherche Scientifique (SPD FRS‐FNRS). Rik Vandenberghe's institution has clinical trial agreements (RV as site PI) with Alector, BMS, Denali, Eli Lilly, J&J, UCB. RV's institution has consultancy agreements (RV as DSMB or DMC member) with ACImmune and Novartis. Bernard Hanseeuw has received consulting fees from Biogen, Eisai, Roche, and Eli Lilly (paid to institution) over the past 5 years. Sebastiaan Engelborghs has received consulting fees from Biogen (paid to institution), Eisai (paid to institution), Icometrix (paid to institution), Janssen (paid to institution), Eli Lilly, Novartis (paid to institution), Remynd (paid to institution). S.E. holds patent EP3452830B1 for an assay for the diagnosis of a neurological disease (licenced to ADX) (Neurosciences NV & Euroimmun Medizinische Labordiagnostika AG). S.E. is a member of SMB/SAB for EU‐H2020 project RECAGE (unpaid) and is chairman of the DSMB of PRImus‐AD (paid to institution). Olivier Deryck has received consulting fees from Biogen, Roche, Eisai and Novartis. The other authors declare no conflicts of interest.

## Data Availability

Data sharing not applicable to this article as no datasets were generated or analysed during the current study.
